# XY0206: A promising sunitinib-derived FLT3 inhibitor targeting therapy-resistant acute myeloid leukemia

**DOI:** 10.1172/JCI212370

**Published:** 2026-10-15

**Authors:** Yiping Yang

The management of FLT3-mutated acute myeloid leukemia (AML) remains constrained by the emergence of resistance to approved FLT3 inhibitors, necessitating continued therapeutic innovation. In this issue, Shen, Yang, Xu, and colleagues present a comprehensive characterization of XY0206, a structurally optimized derivative of sunitinib, demonstrating its capacity to target multiple FLT3-dependent resistance states in AML (1).

Sunitinib is a clinically approved multikinase inhibitor for solid tumors with potent activity against several receptor tyrosine kinases, including FLT3. However, its broad kinase profile contributes to off-target toxicities, limiting its applications in hematologic malignancies. Through integrated biochemical, multi-omics, and functional analyses, the authors demonstrated that the sunitinib-derived XY0206 preferentially suppressed FLT3-centered signaling networks while retaining activity against clinically challenging resistance states, including the F691L gatekeeper mutation that confers cross-resistance to currently approved FLT3 inhibitors (2). The clinical relevance is underscored by a phase I/II trial showing a 45.7% composite complete remission rate overall, with 60.0% in patients with FLT3 internal tandem duplication mutation, including responses in patients previously exposed to FLT3 inhibitors (1).

The research question addressed here is compelling because FLT3 inhibitor resistance represents a major therapeutic obstacle in AML. Resistance mechanisms are increasingly understood not as uniform loss of target dependence, but as adaptive states where residual FLT3 signaling persists through microenvironment-derived survival signals, on-target mutations, or off-target genetic alterations (3, 4). The F691L gatekeeper mutation is particularly problematic, and few agents have demonstrated meaningful activity against this alteration (5). XY0206’s preserved activity in this context, supported by molecular docking and cellular thermal shift assays demonstrating stable engagement with both wild-type and mutant FLT3, represents a significant advance (1).

The implications for future work are substantial. First, the graded model of FLT3 dependency emerging from this study suggests that patient selection strategies should move beyond binary mutation status to incorporate functional assessments of FLT3 pathway engagement. Second, the observation that downstream RAS pathway activation (as seen in Shen, Yang, Xu et al.’s GilR2 model with NRAS mutation; ref. 1) diminishes XY0206 sensitivity highlights the need for combination strategies targeting both FLT3 and parallel resistance pathways (6). Third, the ongoing phase III trial (NCT05876832) will be crucial to define XY0206’s clinical benefit relative to approved inhibitors and identify molecular correlates of response.

While their limitations include limited F691L-positive patient material and the need for direct pharmacodynamic assessment in patients, the study by Shen, Yang, Xu, and colleagues (1) establishes XY0206 as a mechanistically differentiated agent targeting multiple FLT3-dependent resistance states. This positions XY0206 as a promising therapeutic option for patients with FLT3-mutated AML who have exhausted current treatment options, particularly those with reduced sensitivity to existing FLT3 inhibitors.

## Conflict of interest

The author has declared that no conflict of interest exists.

## Version 1 10/15/2026

Electronic publication
